# Reduction in mitochondrial DNA methylation leads to compensatory increase in mitochondrial DNA content: novel blood-borne biomarkers for monitoring occupational noise

**DOI:** 10.1265/ehpm.25-00006

**Published:** 2025-05-24

**Authors:** Jia-Hao Yang, Zhuo-Ran Li, Zhuo-Zhang Tan, Wu-Zhong Liu, Qiang Hou, Pin Sun, Xue-Tao Zhang

**Affiliations:** 1 Department of Occupational Health & Toxicology, School of Public Health, Fudan University, Shanghai, China; 2 Eye & ENT Hospital, Fudan University, Shanghai, China; 3 Shanghai Institute of Occupational Disease for Chemical Industry (Shanghai Institute of Occupational Safety & Health), Shanghai, China

**Keywords:** Occupational noise exposure, Hearing abnormality, Mitochondrial DNA damage, Mitochondrial DNA methylation

## Abstract

**Background:**

Prolonged occupational noise exposure poses potential health risks, but its impact on mitochondrial DNA (mtDNA) damage and methylation patterns remains unclear.

**Method:**

We recruited 306 factory workers, using average binaural high-frequency hearing thresholds from pure-tone audiometry to assess noise exposure. MtDNA damage was evaluated through mitochondrial DNA copy number (mtDNAcn) and lesion rate, and mtDNA methylation changes were identified via pyrophosphate sequencing.

**Results:**

There was a reduction in MT-RNR1 methylation of 4.52% (95% CI: −7.43% to −1.62%) among workers with abnormal hearing, whereas changes in the D-loop region were not statistically significant (*β* = −2.06%, 95% CI: −4.44% to 0.31%). MtDNAcn showed a negative association with MT-RNR1 methylation (*β* = −0.95, 95% CI: −1.23 to −0.66), while no significant link was found with D-loop methylation (*β* = −0.05, 95% CI: −0.58 to 0.48). Mediation analysis indicated a significant increase in mtDNAcn by 10.75 units (95% CI: 3.00 to 21.26) in those with abnormal hearing, with MT-RNR1 methylation mediating 35.9% of this effect.

**Conclusions:**

These findings suggest that occupational noise exposure may influence compensatory increases in mtDNA content through altered MT-RNR1 methylation.

**Supplementary information:**

The online version contains supplementary material available at https://doi.org/10.1265/ehpm.25-00006.

## Graphical Abstract

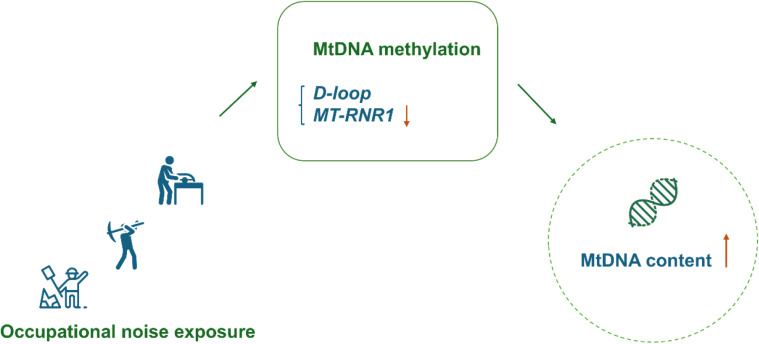

## 1. Introduction

Noise is a well-known contributor to various adverse health outcomes. Recent estimates suggest that around 32.6 million workers in China face occupational noise exposure [1]. Such exposure can cause irreversible auditory damage, leading to noise-induced hearing loss. Moreover, there is growing evidence linking chronic noise exposure to non-auditory health risks, including cardiovascular and metabolic disorders, cognitive impairments, mental health issues, sleep disturbances, and reproductive challenges [2–7]. Despite these associations, the precise pathogenic mechanisms of noise remain unclear.

Noise exposure assessment typically relies on calculating cumulative exposure through environmental assessments in workplaces. However, this method has limitations, as sampling equipment can be easily dislodged by worker movement and is affected by inconsistent earplug use, both in terms of duration and compliance with standardized practices [8, 9]. Consequently, environmental noise measurements may not accurately reflect actual in-ear noise exposure, introducing potential bias. Numerous epidemiological studies have shown a strong link between high-frequency hearing loss and occupational noise exposure [9, 10]. The average bilateral high-frequency threshold level can serve as a biomarker for cumulative exposure, indicating noise intensity and duration [11–13].

Mitochondria are vital organelles involved in numerous cellular activities such as energy production, reactive oxygen species generation, cellular metabolism, and apoptosis. Mitochondria contain their own maternally inherited genomes, known as mitochondrial DNA (mtDNA). Unlike nuclear DNA, mtDNA is circular, double-stranded, and particularly susceptible to oxidative stress due to its lack of histone protection and proximity to the electron transport chain. MtDNA damage can be indicated by its copy number (mtDNAcn), with changes in mtDNAcn being a potential biomarker of mitochondrial dysfunction [14]. Notably, Chen et al. found that noise exposure significantly reduced mtDNAcn levels in rats’ cochleae [15]. Our previous study also observed a significant correlation between cumulative noise exposure and decreased peripheral blood mtDNAcn among male workers [16]. Nevertheless, mtDNAcn is an indirect biomarker of mitochondrial function, and a direct measure of mtDNA damage is lacking. Long-run Real-time DNA Damage Quantification PCR (LORD-Q PCR) can specifically target mitochondrial gene segments, providing data on mtDNA integrity and damage fragments [17–19]. We, therefore, used mtDNA lesion rate via LORD-Q PCR to quantify direct mtDNA damage levels.

DNA methylation is a well-studied epigenetic mechanism, with increasing research suggesting that environmental exposure can alter mtDNA methylation [20, 21]. Altered mtDNA methylation has been associated with clinical conditions such as cardiovascular and neurodegenerative diseases, tumors, and aging [20, 22, 23]. Some studies also report a significant correlation between mtDNA methylation levels and mtDNAcn [20]. While noise as an environmental stressor can modify nuclear DNA methylation [24–26], it is unclear whether noise exposure affects mtDNA methylation and if such modifications contribute to noise-induced health effects.

To address this gap, we investigated the impact of occupational noise exposure on mtDNA damage and epigenetic regulatory mechanisms. Our study involved participants from a manufacturing plant in Shanghai, China, who were exposed to occupational noise. We assessed mitochondrial functional impairment using mtDNAcn and mtDNA integrity, while hearing abnormality, defined by average binaural high-frequency hearing thresholds, served as a biomarker of noise exposure. Additionally, we conducted mediation analyses to explore whether mtDNA methylation mediates the effect of noise exposure on mtDNAcn.

## 2. Methods

### 2.1. Study population

Employees from a manufacturing company in Shanghai who had been exposed to occupational noise for at least one year were recruited for this study. The control group consisted of non-noise-exposed managers from the same company. Workers with a history of long-term medication usage or recent medical radiation testing within the last two weeks were excluded, as were those with pre-existing cardiovascular, endocrine, tumor, auditory system, or genetic illnesses, as well as those with a familial history of deafness. A total of 306 workers participated in the study after providing informed consent, completing questionnaires, donating blood samples, and undergoing occupational health examinations.

Early in the morning, in the fasting state, 5 ml of venous blood was collected from each worker by professional nurses. The blood samples were then coated with EDTA anticoagulant and stored at −80 °C. Our study was approved by the Ethics Committee of Shanghai Institute of Occupational Safety & Health (IRB approval number: IRB#EC-2024-02).

### 2.2. Assessment of hearing abnormality

Participants were classified as having occupational noise exposure if they had been exposed to noise with an equivalent continuous A-weighted sound pressure level (LAeq) of ≥80 dB(A) for at least one year [27, 28]. This classification was verified through occupational hazard monitoring reports from authoritative organisations, ensuring participants met the criteria of LAeq ≥80 dB(A) and a minimum one-year exposure duration. The control group consisted of managers from the same facility, who were exposed to LAeq levels of <80 dB(A) in their work environment.

Electro audiometry was conducted at least 12 hours after participants completed their last shift to ensure accurate results. Hearing tests were administered by qualified medical personnel using a pure tone audiometer (model AD229b, Interacoustics, Denmark). In accordance with the “Diagnosis of Occupational Noise-Induced Deafness” standards in China (GBZ 49-2014), hearing abnormality was defined as a binaural high-frequency average hearing threshold exceeding 25 dB in workers.

### 2.3. Assessment of mitochondrial DNA (mtDNA) content

As previously reported [16], using a PCR instrument (Thermo Scientific, America), the mtDNA content was determined by quantitative PCR (qPCR). Expressed as relative mtDNAcn with the ratio between a target gene (mtND1) to a nuclear gene (hbg), the mtDNA content was calculated: $\text{MtDNA content}=2^{-(Ct_{\text{ND}1}-Ct_{\text{HBG}})}$ . All samples were run in triplicates for both mtND1 and hbg (Primer sequence information see in Table S1) in a run with a 96-well plate. The PCR reaction components for each well are shown in Table S2. To control quality, only PCR amplification efficiencies between 90% and 110% and triple standard deviation <0.25 were accepted for each run.

### 2.4. MtDNA integrity assay

As previously reported by Szabó [19], we performed LORD-Q PCR to detect mtDNA integrity using a kit called G5 High-Fidelity DNA Polymerases (EnzyArtisan, Shanghai) together with a fluorescence qPCR instrument (Thermo Scientific, America). Briefly, the lesion rate per 10 kb of mtDNA is calculated by measuring the Ct values of long and short fragment amplicons using an undamaged DNA sample as a reference. The formula is shown below [18]:

$$\begin{array}{rl} & \text{Lesion rate (per 10}\;\text{kb DNA)}\\ & \;=\left[{\left(\frac{\frac{E_{L}{,}^{\text{CpL}}}{E_{S}{,}^{\text{CpS}}}}{(\frac{E_{L}{,}^{\text{CpL}}}{E_{S}{,}^{\text{CpS}}}{)}_{\text{Ref}}}\right)}^{\left(\frac{1}{{\text{length}}_{L}}\right)}-1\right]*10000 \end{array}$$

For primer synthesis, we selected the long fragment of mitochondrial DNA (3724bp) and the short fragment (50bp) that have been validated by Dannenmann [18] (see Table S1). Details of the PCR reaction components and procedure are provided in Table S3 and Table S4, respectively.

### 2.5. Analysis of mtDNA methylation

A DNA extraction kit (Lifefeng Biotechnology Co, Shanghai, China) was used to extract genomic DNA from peripheral blood. Following extraction, 500 ng of DNA was bisulfite-treated using EZ DNA Methylation Kit (Zymo Research, Irvine, CA, USA). We selected two sections of mtDNA, namely *D-loop* and *MT-RNR1*, which had been previously confirmed as reliable [29, 30]. Using a PCR device (Eppendorf, Germany), the regions of mtDNA were amplified by PCR with 2×Taq PCR Master Mix (Lifefeng Biotechnology Co, Shanghai, China), and information on primers were detailed in Table S5.

Using PyroMark Q24 instrument (Qiagen, Germany), the mtDNA products were sequenced after PCR amplification. For *D-loop* and *MT-RNR1*, the methylation level was measured as the percentage of methylated cytosines over the sum of methylated and unmethylated cytosines.

### 2.6. Covariates

We collected demographic data, including age, sex, and BMI, as well as lifestyle factors such as smoking and drinking status. Individuals with a smoking history of at least six months were categorized as smokers, while those who consumed alcohol at least once a week in the past year were classified as drinkers. Based on the workers’ environmental characteristics and existing literature [8, 16, 31], age, sex, BMI, smoking status, and drinking status were selected as covariates for our analysis.

### 2.7. Statistical analysis

Statistical analyses were conducted using R version 4.4.1. A two-tailed test with a p-value of less than 0.05 was considered statistically significant. For skewed continuous variables, results were presented as medians and interquartile ranges (IQR), while categorical variables were summarized as numbers (N) and percentages (%).

Generalized linear models were employed to investigate the associations between occupational noise exposure, mtDNA damage, and mtDNA methylation. Hearing abnormality was used as a biomarker indicator of occupational noise exposure. Two methods were employed for sensitivity analyses. First, we included a continuous variable, the binaural high-frequency average hearing threshold, in the regression analyses of noise-exposed workers to evaluate the stability of the exposure subgroups. Second, we conducted Bayesian regression analyses, a statistical approach well-suited for studies with small sample sizes, to assess the robustness of the relationships among noise exposure, mtDNA damage, and mtDNA methylation.

To further explore the mediating role of mtDNA methylation in the relationship between occupational noise exposure and mtDNAcn, mediation analyses were conducted using the *mediation* package in R, reporting average causal mediation effects (ACME), average direct effects (ADE), total effects, and the proportion mediated. Additionally, bias-corrected and accelerated (BCa) bootstrap resampling with 2,000 iterations was implemented to estimate 95% confidence intervals for the effect estimates.

## 3. Results

### 3.1. Characteristics of participants

Table 1 summarizes the demographic characteristics of the study participants. Of the 306 workers, 43.5% were male, with a median age of 35.00 years [IQR: 31.00, 40.00] and an age range of 21.00 to 49.00 years.

**Table 1:** Baseline characteristics of participants.

**Variables**	**Exposed, normal hearing**	**Exposed, abnormal hearing**	**Non-exposed**	***P value***
Participants, N	255	22	29	
Age, years, median [IQR]	33.00 [30.00, 39.00]	38.00 [33.00, 41.00]	42.00 [37.00, 44.00]	<0.001
Male, n (%)	114 (44.7)	16 (72.7)	3 (10.3)	<0.001
BMI^a^, kg/m^2^, median [IQR]	22.00 [20.20, 24.40]	22.00 [20.70, 23.85]	22.70 [21.15, 24.00]	0.804
Current smoker, n (%)	31 (12.2)	3 (13.6)	0 (0.0)	0.132
Current drinker, n (%)	34 (13.3)	4 (18.2)	0 (0.0)	0.083
**MtDNA damage**				
Relative mtDNAcn, median [IQR]	28.38 [23.24, 35.79]	32.57 [22.06, 42.69]	35.34 [24.36, 42.03]	0.084
Detected mtDNA lesion rate^b^, 10 kb, median [IQR]	8.47 [4.20, 10.65]	9.34 [5.49, 12.91]	9.57 [6.17, 12.87]	0.093
**MtDNA methylation**				
*D-loop*-average^c^, %, median [IQR]	4.19 [3.09, 5.71]	3.96 [3.26, 4.79]	5.30 [3.68, 7.16]	0.067
*D-loop-Pos.1*, %, median [IQR]	3.77 [2.73, 5.36]	3.51 [2.51, 3.87]	5.42 [3.22, 6.75]	0.031
*D-loop-Pos.2*, %, median [IQR]	5.67 [4.44, 7.15]	5.19 [4.64, 6.15]	6.35 [4.87, 8.63]	0.171
*D-loop-Pos.3*, %, median [IQR]	3.30 [2.36, 5.29]	3.19 [2.19, 4.32]	4.34 [2.88, 6.84]	0.134
*MT-RNR1-average*^d^, %, median [IQR]	13.74 [11.39, 17.59]	11.54 [9.83, 12.85]	14.74 [12.61, 17.78]	0.007
*MT-RNR1-Pos.1*, %, median [IQR]	12.34 [10.22, 15.48]	9.80 [8.18, 11.18]	13.01 [10.84, 14.94]	0.002
*MT-RNR1-Pos.2*, %, median [IQR]	15.09 [12.67, 19.37]	13.53 [11.37, 14.86]	16.48 [13.36, 20.18]	0.026

**Abbreviation:** BMI, body mass index; mtDNA, mitochondrial DNA; mtDNAcn, mitochondrial DNA copy number.

N ^a^ = 271 for BMI data; N ^b^ = 298 for mtDNA lesion rate data; N ^c^ = 157 for *D-loop* data; N ^d^ = 216 for *MT-RNR1* data.

Among the overall participants, 22 were not exposed to noise, while 277 were exposed. Notably, all non-exposed participants had normal hearing, whereas 22 of the exposed participants exhibited abnormal hearing. Statistically significant differences were observed in age and sex across the three groups, while no significant differences were found in BMI, smoking status, or drinking status.

In the noise-exposed workers, the median mtDNAcn for those with normal hearing was 28.38, compared to 32.57 for those with abnormal hearing; and the mtDNA lesion rates were 8.47 versus 9.34 lesions per 10 kb, respectively. Additionally, pyrophosphate sequencing revealed an average D-loop methylation level of 4.19% for participants with normal hearing and 3.96% for those with abnormal hearing. The MT-RNR1 methylation levels were 13.74% for the normal hearing group and 11.54% for the abnormal hearing group.

### 3.2. Effects of noise exposure on mtDNA methylation

Table 2 outlines the impact of occupational noise exposure on mtDNA methylation in the D-loop and MT-RNR1 regions. In the D-loop non-coding region, there was no significant decrease in methylation levels in the noise-exposed group with abnormal hearing compared to the group with normal hearing (*β* = −2.06%, 95% CI: −4.44% to 0.31%; *P* = 0.091). In contrast, for MT-RNR1, the methylation level in the noise-exposed group with abnormal hearing was reduced by 4.52% compared to the normal hearing group (95% CI: −7.43% to −1.62%; *P* = 0.003). Sensitivity analyses confirmed the robustness of these results (Tables S6–S7).

**Table 2:** Associations between hearing abnormality and mtDNA methylation levels.

**MtDNA methylation levels, %**	**Exposure biomarker**	**N**	***β (95% CI)***	***P value***
*D-loop*				
Model 1	Exposed, normal hearing	116	Ref.	
	Exposed, abnormal hearing	17	−2.14 (−4.50, 0.22)	0.078
	Non-exposed	24	0.20 (−1.80, 2.19)	0.847
Model 2	Exposed, normal hearing	116	Ref.	
	Exposed, abnormal hearing	17	−2.06 (−4.44, 0.31)	0.091
	Non-exposed	24	0.19 (−1.82, 2.19)	0.856
*MT-RNR1*				
Model 1	Exposed, normal hearing	167	Ref.	
	Exposed, abnormal hearing	20	−4.54 (−7.43, −1.65)	0.002
	Non-exposed	29	−0.98 (−3.34, 1.38)	0.418
Model 2	Exposed, normal hearing	167	Ref.	
	Exposed, abnormal hearing	20	−4.52 (−7.43, −1.62)	0.003
	Non-exposed	29	−1.08 (−3.46, 1.29)	0.371

**Abbreviation:** Ref., reference; mtDNA, mitochondrial DNA; mtDNAcn, mitochondrial DNA copy number.

Model 1 was adjusted for age, sex and BMI.

Model 2 was adjusted for age, sex, BMI, smoking status and drinking status.

### 3.3. Association between noise exposure and mtDNA damage biomarkers

Then, mtDNAcn and mtDNA lesion rate were employed as biomarkers to evaluate mtDNA damage (Table 3). After adjusting for potential confounders, the analysis showed a trend toward an increased mtDNAcn in the noise-exposed group with abnormal hearing compared to those with normal hearing (*β* = 8.10, 95% CI: 1.96 to 14.24; *P* = 0.010).

**Table 3:** Associations between hearing abnormality and mtDNA damage.

**MtDNA damage indicators**	**Exposure biomarker**	**N**	***β (95% CI)***	***P value***
Relative mtDNAcn				
Model 1	Exposed, normal hearing	255	Ref.	
	Exposed, abnormal hearing	22	8.03 (1.92, 14.14)	0.011
	Non-exposed	29	7.29 (2.12, 12.46)	0.006
Model 2	Exposed, normal hearing	255	Ref.	
	Exposed, abnormal hearing	22	8.10 (1.96, 14.24)	0.010
	Non-exposed	29	7.42 (2.23, 12.61)	0.005
Detected mtDNA lesion rate, 10 kb				
Model 1	Exposed, normal hearing	249	Ref.	
	Exposed, abnormal hearing	20	2.41 (−0.02, 4.84)	0.053
	Non-exposed	29	1.24 (−0.72, 3.19)	0.217
Model 2	Exposed, normal hearing	249	Ref.	
	Exposed, abnormal hearing	20	2.21 (−0.21, 4.64)	0.075
	Non-exposed	29	1.28 (−0.67, 3.23)	0.200

**Abbreviation:** Ref., reference; mtDNA, mitochondrial DNA; mtDNAcn, mitochondrial DNA copy number.

Model 1 was adjusted for age, sex and BMI.

Model 2 was adjusted for age, sex, BMI, smoking status and drinking status.

Regarding the mtDNA lesion rate, after controlling for confounders, no significant increase was detected in the noise-exposed group with abnormal hearing when compared to the normal hearing group (*β* = 2.21, 95% CI: −0.21 to 4.64; *P* = 0.075). Sensitivity analyses confirmed the robustness of these results (Tables S6–S7).

### 3.4. Mediation effect of methylation on the association between noise exposure and mtDNAcn

The association between mtDNA methylation and mtDNAcn is summarized in Table 4. After adjusting for potential confounders, a 1% increase in MT-RNR1 methylation corresponded to a decrease of 0.95 units in mtDNAcn (95% CI: −1.23, −0.66; *P* < 0.001). No significant association was found between mtDNAcn and *D-loop* region methylation levels (*β* = −0.05, 95% CI: −0.58, 0.48; *P* = 0.859), making only the *MT-RNR1* gene suitable for mediation analysis. Sensitivity analyses confirmed the robustness of the relationships between mtDNAcn and mtDNA methylation (Table S8).

**Table 4:** Associations between mtDNA methylation and mtDNAcn.

**MtDNA methylation, %**	**N**	**MtDNAcn**	

		***β (95% CI)***	***P value***
*D-loop*	157		
Model 1		−0.06 (−0.59, 0.46)	0.812
Model 2		−0.05 (−0.58, 0.48)	0.859
*MT-RNR1*	216		
Model 1		−0.96 (−1.24, −0.68)	<0.001
Model 2		−0.95 (−1.23, −0.66)	<0.001

**Abbreviation:** Ref., reference; mtDNA, mitochondrial DNA; mtDNAcn, mitochondrial DNA copy number.

Model 1 was adjusted for age, sex and BMI.

Model 2 was adjusted for age, sex, BMI, smoking status and drinking status.

Table 5 presents the mediation analysis of *MT-RNR1* methylation in the relationship between occupational noise exposure and mtDNAcn. The analysis revealed significant total (*β* = 10.75, 95% CI: 3.00, 21.26; *P* = 0.010) and indirect (*β* = 3.86, 95% CI: 2.14, 6.24; *P* < 0.001) effects. *MT-RNR1* methylation mediated 35.9% (95% CI: 13.8%, 57.0%; *P* = 0.010) of the effect of occupational noise exposure on mtDNAcn.

**Table 5:** Mediation analysis of occupational noise exposure affecting mtDNAcn via *MT-RNR1* methylation.

**Mediation effect via ** ***MT-RNR1* methylation**	**N**	***β (95% CI)***	***P value***
Effect	182		
ACME		3.86 (2.14, 6.24)	<0.001
ADE		6.89 (−0.09, 16.46)	0.072
Total effect		10.75 (3.00, 21.26)	0.010
Mediated proportion		35.9% (13.8%, 57.0%)	0.010

**Abbreviation:** Ref., reference; mtDNA, mitochondrial DNA; mtDNAcn, mitochondrial DNA copy number; ACME, average causal mediation effect; ADE, average direct effect. Model was adjusted for age, sex, BMI, smoking status and drinking status.

## 4. Discussion

In this study, we identified a marked reduction in MT-RNR1 methylation levels among individuals with hearing abnormalities compared to those with normal auditory function. Additionally, we found that decreased MT-RNR1 methylation was associated with an increase in mtDNAcn. Most importantly, we observed that workers exposed to occupational noise exhibited a compensatory increase in mtDNA content through reduced MT-RNR1 methylation, with a mediating effect of 35.9%.

Given that China has one of the largest labor forces globally, with a substantial segment exposed to occupational noise, this study holds significant importance. We employed average binaural high-frequency hearing thresholds to identify individuals with hearing abnormalities, establishing this as a biomarker for internal exposure to occupational noise. Numerous epidemiological studies have demonstrated that high-frequency hearing notches at 3000, 4000, or 6000 Hz are indicative of noise-induced hearing loss and may reflect the damage response of cochlear hair cells to cumulative noise exposure [13, 32]. Moreover, bilateral high-frequency average hearing loss is closely linked to cumulative occupational noise exposure, as workers typically experience binaural noise.

It is important to note that while the cochlea is the target organ for hearing abnormalities, all mitochondria-related indicators measured in this study were derived from peripheral blood samples. In addition to the health risks to the auditory system, noise acts as an environmental stressor, inducing a systemic stress response via the hypothalamic-pituitary-adrenal axis and the central nervous system [33, 34]. Research has indicated that respiratory chain function is severely impaired in patients with high levels of mtDNA variants, while mtDNAcn is significantly elevated, suggesting a compensatory alteration in cellular respiratory function through increased mtDNA replication [35]. Therefore, in the present study, the observed increase in mtDNAcn is more likely a compensatory response to systemic stress. Future animal studies are necessary to confirm whether mtDNAcn can serve as a reliable marker for hearing abnormalities by observing changes in mtDNAcn within the cochlea, the target organ.

In this study, we documented an increase in mtDNAcn in a noise-exposed population with hearing abnormalities. MtDNA is particularly vulnerable to damage from oxidative stress due to its lack of introns, histones, and limited repair mechanisms [36]. Mild oxidative stress may lead to an adaptive increase in mtDNAcn as a compensatory response to impaired mitochondrial respiration. Conversely, prolonged oxidative stress can disrupt mitochondrial membrane permeability and cause the release of pro-apoptotic proteins, ultimately leading to a decrease in mtDNAcn [37, 38]. Martin Picard has suggested that variations in blood mtDNAcn may reflect complex hematopoietic and immune processes in the circulatory system [39]. Overall, mitochondria can influence T cell activation and proliferation by producing reactive oxygen species (ROS) [40]. Thus, our findings imply that noise exposure may trigger adaptive changes in the circulatory system to support mitochondrial respiratory function, particularly pronounced in individuals with hearing abnormalities.

Despite mtDNA methylation levels being lower than those of nuclear DNA, alterations in mtDNA methylation have been linked to various diseases, including cancer, cardiovascular diseases, metabolic disorders, and neuropsychiatric conditions. Various environmental factors have also been shown to influence mtDNA methylation levels [20–22, 41]. An animal study has suggested that low-intensity environmental noise exposure affects DNA methylation levels in the rat brain [42]. Furthermore, it has been reported that physiological or psychological stressors can impact normal epigenetic patterns [43]. Noise, as a chronic environmental stressor, has the potential to cause alterations in mtDNA methylation. The D-loop, a non-coding region of mtDNA, serves as a critical hotspot for epigenetic regulation, significantly influencing mtDNA replication and transcription [29]. However, we found no significant difference in D-loop methylation levels between participants with hearing abnormalities and those with normal hearing.

Our study observed reduced levels of MT-RNR1 methylation in workers with hearing abnormalities, suggesting that MT-RNR1 methylation may mediate the compensatory regulation of mtDNAcn in response to noise exposure. MT-RNR1, a mitochondrial gene encoding a 12S rRNA protein, is crucial for the proper function and integrity of mitochondrial ribosomes. Structural instability of the encoded protein can adversely affect subsequent translation processes, potentially leading to mitochondrial translation arrest [44, 45]. Novielli et al. observed that increased mitochondrial levels in fetal blood were associated with hypomethylation of the MT-RNR1 gene as a response to oxidative stress [46]. This implies that noise, by reducing MT-RNR1 methylation through systemic stressors, may enhance MT-RNR1 expression and promote 12S rRNA production, thereby supporting normal mitochondrial function. In response to increased respiratory demands for ROS scavenging, mitochondria regulate the augmentation of mitochondrial DNA content via compensatory mechanisms [47]. Our findings are consistent with those of Janssen et al. [29, 48], who also reported a significant negative correlation between MT-RNR1 methylation and mtDNA content. In summary, noise appears to regulate mtDNAcn in response to oxidative stress through mtDNA methylation epigenetic mechanisms. Given the simplicity and ease of measurement of mtDNA methylation and mtDNAcn—both of which are blood-borne indicators—they offer significant potential for monitoring systemic compensatory responses in workers exposed to occupational noise. Future research linking these mitochondrial metrics to noise-induced health risks could be invaluable for disease prevention, although more sophisticated study designs are still necessary.

To our knowledge, this study is the first to explore the response of mtDNA methylation to occupational noise exposure. Our findings reveal, for the first time, a compensatory increase in mtDNA content through the mechanism of mtDNA methylation in noise-exposed workers with hearing abnormalities, contributing to a deeper understanding of the molecular pathogenesis of noise-related health issues. Additionally, we observed a robust association between average binaural high-frequency hearing thresholds and both mtDNA methylation and mtDNAcn in noise-exposed workers, as validated through sensitivity analyses. There remains a scarcity of studies on biomarkers of noise exposure, and our research may provide new insights for the risk management of populations exposed to occupational noise.

Despite these contributions, our study does have limitations. First, the cross-sectional design restricts our ability to establish causal relationships. Furthermore, all mtDNA-related indicators in this study were sourced from blood samples, while the cochlea remains the target organ for hearing abnormalities. Future zoological studies are necessary to further elucidate the relationship between auditory systemic effects and mtDNAcn. Nevertheless, we implemented stringent exclusion criteria, standardized questionnaires, and comprehensive occupational health assessments to minimize potential confounders and biases in our statistical analyses. Secondly, we were unable to obtain mtDNA methylation data for all samples due to limitations in the number of available DNA samples and quality control issues with the methylation assays. This limitation may have hindered our ability to definitively determine a dose-response relationship between noise exposure and D-loop methylation levels. Nonetheless, the majority of our results remained statistically significant despite the relatively small sample size. Additionally, a single measurement of mtDNA methylation may not capture intra-individual changes over time. While mtDNA damage and methylation biomarkers in blood are relatively straightforward and easy to measure, further studies are warranted to explore their association with health effects induced by occupational noise. Such research could yield valuable insights for enhancing occupational noise health surveillance. Addressing these limitations will be crucial for promoting effective health risk management in populations exposed to occupational noise.

## 5. Conclusion

Our study indicates that occupational noise exposure may decrease MT-RNR1 methylation, with MT-RNR1 methylation partially mediating noise-induced changes in mtDNAcn. To validate these findings and deepen our understanding of the molecular mechanisms involved, future longitudinal studies with larger sample sizes are essential, focusing on the role of epigenetic modifications in noise-related pathogenic processes.

## Supplementary information

**Additional file 1:** **Table S1.** Primer sequence information for mtDNA content and mtDNA integrity assay. **Table S2.** PCR reaction components for mtDNA content assay. **Table S3.** PCR reaction components for mtDNA integrity assay. **Table S4.** PCR reaction procedure for mtDNA integrity assay. **Table S5.** Bisulfite-Pyrosequencing primer sequence information. **Table S6.** Associations between binaural mean hearing threshold and mtDNA methylation and damage. **Table S7.** Associations between hearing abnormality and mtDNA methylation and damage using Bayesian linear models. **Table S8.** Association between mtDNA methylation and mtDNAcn using Bayesian linear models.

## Declarations

### Availability of data and material

The data in this study are available from the corresponding author upon reasonable request.
