# Supplementary material for: Reduction in mitochondrial DNA methylation leads to compensatory increase in mitochondrial DNA content: novel blood-borne biomarkers for monitoring occupational noise
Source: Environ Health Prev Med. 2025 May 24;30:40. doi: 10.1265/ehpm.25-00006 (PMC12127082; doi:10.1265/ehpm.25-00006)
Supplement: Supplementary file 1 — Additional file 1: Table S1. Primer sequence information for mtDNA content and mtDNA integrity assay. Table S2. PCR reaction components for mtDNA content assay. Table S3. PCR reaction components for mtDNA integrity assay. Table S4. PCR reaction procedure for mtDNA integrity assay. Table S5. Bisulfite-Pyrosequencing primer sequence information. Table S6. Associations between binaural mean hearing threshold and mtDNA methylation and damage. Table S7. Associations between hearing abnormality and mtDNA methylation and damage using Bayesian linear models. Table S8. Association between mtDNA methylation and mtDNAcn using Bayesian linear models. [file ehpm-30-040-s001.docx]

**Reduction in mitochondrial DNA methylation leads to compensatory increase in mitochondrial DNA content: Novel blood-borne biomarkers for monitoring occupational noise**

**Supporting Information**

Jia-Hao Yang ^a,1^, Zhuo-Ran Li ^a,1^, Zhuo-Zhang Tan ^b, 1^, Wu-Zhong Liu ^c^, Qiang Hou ^c^, Pin Sun ^a,^ *, Xue-Tao Zhang ^c,^ *

^a^ Department of Occupational Health & Toxicology, School of Public Health, Fudan University, Shanghai, China

^b^ Eye & ENT Hospital, Fudan University, Shanghai, China

^c^ Shanghai Institute of Occupational Disease for Chemical Industry (Shanghai Institute of Occupational Safety & Health), Shanghai, China

^*^ Co-corresponding authors. Department of Occupational Health & Toxicology, School of Public Health, Fudan University, Building 8, No.130 Dong'an Road, Xuhui District, Shanghai 200032, China (Tel NO. +86-021-54237053).

*E-mail address*: [pinsun@fudan.edu.cn (Pin](mailto:pinsun@fudan.edu.cn%20(Pin) Sun), 13818873296@163.com (Xue-Tao Zhang).

^1^ Jia-Hao Yang, Zhuo-Ran Li, and Zhuo-Zhang Tan contributed equally to this work.

**Summary of the supporting information:**

Supporting Information file contains 10 pages and 8 tables.

**Reduction in mitochondrial DNA methylation leads to compensatory increase in mitochondrial DNA content: Novel blood-borne biomarkers for monitoring occupational noise**

**Supporting Information**

**Contents**

Table S1. Primer sequence information for mtDNA content and mtDNA integrity assay.…………………………………………………………………………………S3

Table S2. PCR reaction components for mtDNA content assay.……………………S4

Table S3. PCR reaction components for mtDNA integrity assay...…………………S5

Table S4. PCR reaction procedure for mtDNA integrity assay.………………………S6

Table S5. Bisulfite-Pyrosequencing primer sequence information.…………………S7

Table S6. Associations between binaural mean hearing threshold and mtDNA methylation and damage…………………….………………….…………………S8

Table S7. Associations between hearing abnormality and mtDNA methylation and damage using Bayesian linear models…………………………………………S9

Table S8. Association between mtDNA methylation and mtDNAcn using Bayesian linear models……………………………………………………………………S10

**Table S1**

Primer sequence information for mtDNA content and mtDNA integrity assay.

| Gene | Primer Sequences（5'→3'） | Amplicon（bp） |
| --- | --- | --- |
| mtND1 | F: CACCCAAGAACAGGGTTTGT |  |
|  | R:TGGCCATGGGTATGTTGTTA |  |
| hbg | F: GCTTCTGACACAACTGTGTTCACTAGC |  |
|  | R:CACCAACTTCATCCACGTTCACC |  |
| mtDNA (L) of human | F: ATCGTAGCCTTCTCCACTTC | 3724 |
|  | R: TGGTTAGGCTGGTGTTAGGG |  |
| mtDNA (S) of human | F: GGCCACAGCACTTAAACACA | 50 |
|  | R: TGGTTAGGCTGGTGTTAGGG |  |

**Table S2**

PCR reaction components for mtDNA content assay.

| PCR reaction components | Volumes (μl) |
| --- | --- |
| 2× SYBR GREEN PCR Master MIX | 5 |
| Forward primer（5μM） | 1 |
| Reverse primer（5μM） | 1 |
| DNA template | 1 |
| DNase/Rnase-Free water | 2 |
| Total | 10 |

**Table S3**

PCR reaction components for mtDNA integrity assay.

| PCR reaction components | Volumes |
| --- | --- |
| 2× Diamond PCR Buffer | 10 μl |
| Forward primer（10μM） | 1 μl |
| Reverse primer（10μM） | 1 μl |
| DNA template | 1 μl |
| G5 High-Fidelity DNA Polymerase (1 U/μl) | At least 0.3 μl |
| SYTO® 9 Green-Fluorescent Nucleic Acid Stains (60 μM) | 0.5 μl |
| DNase/Rnase-Free water | Supplemented to a final volume of 20 μl |

**Table S4**

PCR reaction procedure for mtDNA integrity assay.

| Step | Temperature (℃) | Time |  |  |
| --- | --- | --- | --- | --- |
| Pre-denaturation | 95 | 3min |  |  |
| Denaturation | 95 | 15s |  |  |
| Annealing | 60 | 15s |  | 45× |
| Extension | 72 | 135s (long amplicon) /1s (short amplicon) |  |  |

**Table S5**

Bisulfite-Pyrosequencing primer sequence information.

| MtDNA | Primer Sequences（5'→3'） | Amplicon（bp） | Annealing temperature（℃） | Target CpGs |
| --- | --- | --- | --- | --- |
| ***D-loop*** | F: TGGAAAGTGGTTGTGTAGATATTTAA | 139 | 57.4 | 3 |
|  | R: (Biotin) CTTTAATTCCTACCTCATCCTATTATTT |  |  |  |
|  | S: TATGTTYGTTTGTAATATTGAAYGTAGGTGYGAT |  |  |  |
| ***MT-RNR1*** | F: TTTTTAGAGGAGTTTGTTTTGTAAT | 176 | 58.3 | 2 |
|  | R: (Biotin) ATAACCCATTTCTTACCACCTCATA |  |  |  |
|  | S: AGTTTGTTTTGTAAT |  |  |  |

**Table S6**

Associations between binaural mean hearing threshold and mtDNA methylation and damage.

| Binaural mean hearing threshold | Outcome indicators * | N | *β (95% CI)* | *P value* |
| --- | --- | --- | --- | --- |
|  | **MtDNA methylation** |  |  |  |
|  | *D-loop* | 133 | -0.09 (-0.37, 0.18) | 0.497 |
|  | *MT-RNR1* | 187 | -0.39 (-0.75, -0.03) | 0.033 |
|  | **MtDNA damage indicators** |  |  |  |
|  | Relative mtDNAcn | 277 | 0.77 (0.03, 1.50) | 0.041 |
|  | Detected mtDNA lesion rate, 10 kb | 269 | 0.09 (-0.20, 0.38) | 0.546 |

**Abbreviation**: mtDNA, mitochondrial DNA; mtDNAcn, mitochondrial DNA copy number.

* Model was adjusted for age, sex, BMI, smoking status and drinking status.

**Table S7**

Associations between hearing abnormality and mtDNA methylation and damage using Bayesian linear models.

| Outcome indicators * | Exposure biomarker | N | *β (95% CI)* |
| --- | --- | --- | --- |
| **MtDNA methylation** |  |  |  |
| *D-loop* | Exposed, normal hearing | 116 | Ref. |
|  | Exposed, abnormal hearing | 17 | -1.95 (-4.25, 0.32) |
|  | Non-exposed | 24 | 0.16 (-1.84, 2.11) |
| *MT-RNR1* | Exposed, normal hearing | 167 | Ref. |
|  | Exposed, abnormal hearing | 20 | -4.11 (-7.02, -1.25) |
|  | Non-exposed | 29 | -0.97 (-3.16, 1.27) |
| **MtDNA damage** |  |  |  |
| Relative mtDNAcn | Exposed, normal hearing | 255 | Ref. |
|  | Exposed, abnormal hearing | 22 | 5.58 (0.34, 10.82) |
|  | Non-exposed | 29 | 5.65 (1.15, 10.07) |
| Detected mtDNA lesion rate, 10 kb | Exposed, normal hearing | 249 | Ref. |
|  | Exposed, abnormal hearing | 20 | 2.09 (-0.28, 4.52) |
|  | Non-exposed | 29 | 1.23 (-0.73, 3.10) |

**Abbreviation**: Ref., reference; mtDNA, mitochondrial DNA; mtDNAcn, mitochondrial DNA copy number.

* Model was adjusted for age, sex, BMI, smoking status and drinking status.

**Table S8**

Association between mtDNA methylation and mtDNAcn using Bayesian linear models.

| Outcome indicators * | N | MtDNAcn |  |
| --- | --- | --- | --- |
|  |  | *β* | *95% CI* |
| MtDNA methylation, % |  |  |  |
| *D-loop* | 157 | -0.06 | -0.58, 0.46 |
| *MT-RNR1* | 216 | -0.95 | -1.22, -0.67 |

**Abbreviation**: mtDNA, mitochondrial DNA; mtDNAcn, mitochondrial DNA copy number.

* Model was adjusted for age, sex, BMI, smoking status and drinking status.
